# Quantitative distribution of patient-derived leukemia clones in murine xenografts revealed by cellular barcodes

**DOI:** 10.1038/s41375-019-0695-2

**Published:** 2019-12-18

**Authors:** Sabrina Jacobs, Albertina Ausema, Erik Zwart, Ellen Weersing, Maaike J. Kingma, Yasmine A. S. El Menshawi, Gerald de Haan, Leonid V. Bystrykh, Mirjam E. Belderbos

**Affiliations:** 1 Department of Ageing Biology and Stem Cells, European Research Institute for the Biology of Ageing (ERIBA), University Medical Center Groningen (UMCG), University of Groningen, Groningen, The Netherlands; 2 Oncode Institute and Princess Máxima Center for Pediatric Oncology, Utrecht, The Netherlands

**Keywords:** Cancer models, Acute lymphocytic leukaemia, Cancer stem cells

## To the Editor:

Relapse affects ~10% of children diagnosed with acute lymphoblastic leukemia (ALL), and is the leading cause of cancer-related mortality in children, urging for novel diagnostic and therapeutic strategies [1*–*3]. ALL is genetically heterogeneous, consisting of multiple clones with distinct genomic aberrations, which may alter essential cell functions (e.g. proliferation, differentiation, or chemotherapeutic sensitivity) [4]. Furthermore, spatial heterogeneity (i.e. diversity in the localization of ALL clones) may contribute to disease progression [5]. In solid tumors, it is commonly accepted that malignant cells first proliferate at the site of origin, and only later metastasize to distant sites. Here, they may undergo further clonal selection and evolution, resulting in genomic differences between the primary tumor and its metastases [6*–*8]. In contrast, although progenitor B-cell ALL (B-ALL) is thought to originate in the bone marrow, its exact site of origin and patterns of migration are unknown. Previously, we and others found that patient-derived B-ALL clones are asymmetrically distributed in murine xenografts [5, 9]. This skeletal asymmetry is relevant both from a biologic and clinical perspective, as it implies that sampling of a single site may not fully reflect the total body clonal composition, allowing for certain clones to remain undetected. However, as these previous studies only sampled a limited number of anatomic sites and did not include quantitative analysis, the quantitative distribution of leukemia clones across the total body is unknown. To assess the quantitative distribution of leukemia clones, we transplanted barcoded patient-derived B-ALL cells in *Nod/SCID/IL2Rγ*^−/−^ (NSG) mice and determined the leukemia cell content and barcode complexity in individual anatomical locations during different stages of disease (Supplementary methods).

**Fig. 1:**
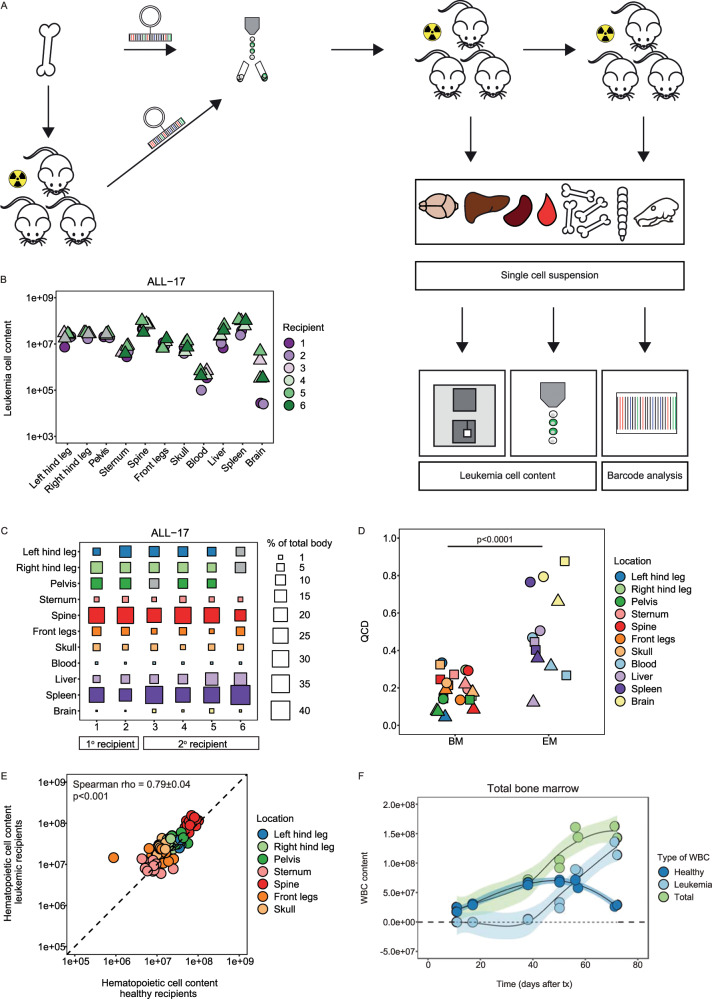
The anatomic distribution of human leukemia cells in murine xenografts is proportional to and limited by the compartment size. **a** Experimental design to quantify the leukemia cell content. Patient-derived bone marrow cells were — directly or derived from primografts — barcoded. Barcoded leukemia cells were sorted for GFP and transplanted into sublethally irradiated NSG mice. ALL-16 and ALL-17 were serially transplanted. Individual locations were analyzed for leukemia cell content and barcode composition. The absolute cell concentration (hematology analyzer) and cell population frequency (flow cytometry) were used to calculate the leukemia cell content. **b** The leukemia cell content in the individual locations of murine xenografts transplanted with ALL-17. Symbols refer to primary (circle) and secondary (triangle) recipients of barcoded leukemia cells. **c** The relative contribution of each anatomical location to the total body leukemia cell content. Gray squares; pelvis of recipient 3 was not sampled and is the average of recipient 4 and 5; hind legs of recipient 6 were analyzed together with pelvis. **d** Quartile coefficient of dispersion (QCD) values, reflecting variability across the xenografts per individual location per patient sample (*n* = 3). QCD values were grouped by bone marrow and extramedullary sites. Each symbol represents a patient sample: ALL-16 (circle), ALL-17 (square), and ALL-19 (triangle). Statistical analysis: two-sided Mann–Whitney *U* test. **e** Correlation between the number of hematopoietic cells in the bone marrow of leukemic (*n* = 16) and healthy (*n* = 4) NSG mice. Hereto, we used a random-comparison model which randomly assigned one out of the four healthy mice to one out of the sixteen leukemic mice to calculate the correlation (*n* = 1000 random comparisons). Data are expressed as mean ± SD. **f** The absolute number of WBCs in the total bone marrow of leukemic mice during disease progression. Distinctions were made between leukemic cells (light blue), healthy murine WBCs (dark blue), and the total number of WBCs (light green). Every dot represents a mouse. Smoothing method ‘loess’ with confidence interval set at 95%. Abbreviations: bone marrow (BM), extramedullary (EM), white blood cells (WBC).

In total, serially transplanted barcoded leukemia cells of three (out of five) patient samples engrafted successfully in 28 recipients (Supplementary Table 1). Using a quantitative method for leukemia-cell detection (Fig. 1a, Supplementary Fig. 1A, B), we showed that at end-stage leukemia, the total murine xenograft harbored 260 × 10^6^ ± 115 × 10^6^ human leukemia cells (mean ± SD of ALL-16, ALL-17, and ALL-19; Supplementary Fig. 2A–C). Of these, 60 ± 15% were located in the bone marrow and 40 ± 15% in extramedullary locations. The leukemia cell content (i.e. the absolute number of leukemia cells) was highly variable between anatomic locations (Fig. 1b, Supplementary Fig. 2D, E), with the consistent observation that the majority of leukemia cells were located in the spine (median: 25%, IQR: 20–30%) and spleen (median: 30%, IQR: 20–40%; Fig. 1c, Supplementary Fig. 2F, G). On the contrary, the blood and pelvis — locations that are routinely sampled for clinical diagnosis and follow-up — only contained a median of respectively 0.06% (IQR: 0.03–0.2%) and 9% (IQR: 8–11%) of the total body leukemia cell content.

We noted that the leukemia cell content in the same bone marrow location was markedly similar across patient samples and xenografts (Fig. 1b, Supplementary Fig. 2D, E). For example, the femur consistently contained 20 × 10^6^ leukemia cells (median of ALL-16, ALL-17, and ALL-19; IQR: 10–30 × 10^6^). In contrast, the leukemia cell content in extramedullary locations was more variable. This variability was quantified by the quartile coefficient of dispersion, which was significantly lower in bone marrow locations (median: 0.20, IQR: 0.15–0.25) compared with extramedullary sites (median: 0.45; IQR: 0.35–0.70, *p* < 0.0001, Fig. 1d). These data indicate that the leukemia cell content in distinct bone marrow sites at end-stage disease is highly predictable, whereas the content in extramedullary sites is more variable.

To address whether the carrying capacity of the bone dictates its leukemia cell content, we compared the number of hematopoietic cells and LSK-SLAM cells in bones of healthy NSG mice with that of mice transplanted with leukemia cells. In total, healthy mice harbored a median of 175 × 10^6^ (IQR: 165 × 10^6^–195 × 10^6^) hematopoietic cells in their bone marrow (Supplementary Fig. 3A). The distribution of these cells across the different bone marrow locations was markedly comparable with the distribution of leukemia cells (Supplementary Fig. 3B). We found a correlation of 0.79 ± 0.04 between the hematopoietic cell distribution in leukemic and healthy mice (Spearman rho, *p* < 0.001, Fig. 1e). Similar observations were made when assessing the more stringently defined population of LSK-SLAM cells (Spearman rho: 0.62 ± 0.03, *p* < 0.001, Supplementary Fig. 3C–E).

If the size of the bone indeed dictates its leukemia cell content, one might postulate that saturation occurs during disease progression. Hereto, we assessed the leukemia cell content across the murine skeleton over time (Supplementary Fig. 4.1A). We demonstrate that the total white blood cell (WBC) content in bone marrow of leukemic mice reached a plateau of ~150 × 10^6^ cells (range 140–160 × 10^6^, Fig. 1f) from 56 days after transplantation. This plateau was primarily due to loss of healthy WBCs throughout disease progression, whereas the leukemia cell content continued to increase over time. Similar patterns were observed when bone marrow locations were analyzed separately (Supplementary Fig. 4.2A–G). In parallel, we observed an exponential increase in the number of splenic leukemia cells, and weight of the spleen (Supplementary Fig. 4.1B–D). Even at late stages of disease, no plateau was reached. Together, this suggests that the size of the bone restricts its leukemia cell content, resulting in competitive loss of healthy WBCs and migration of leukemic blasts to extramedullary sites.

**Fig. 2:**
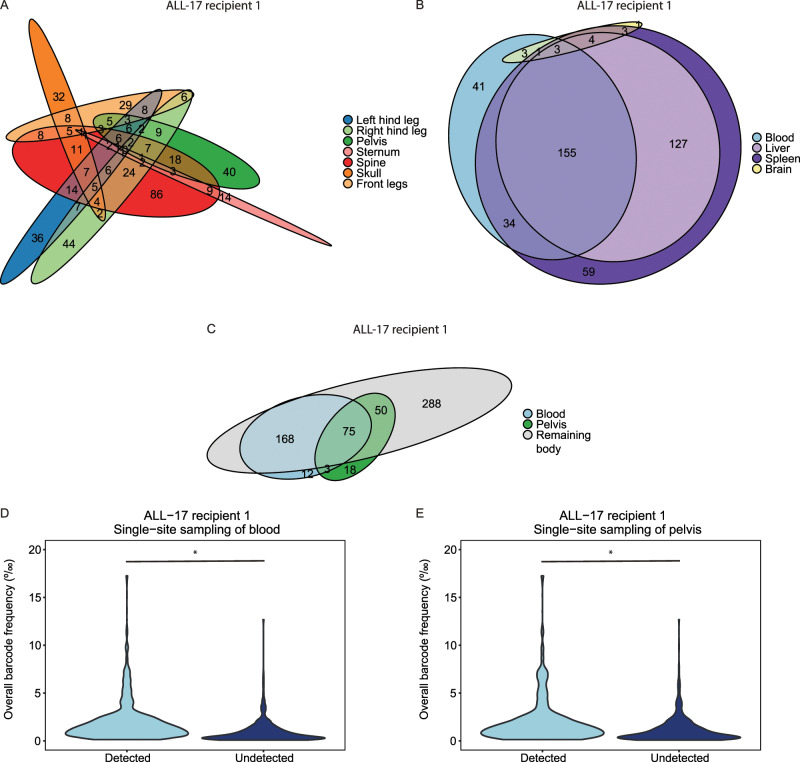
Single-site sampling results in underestimation of total-body leukemia clonal complexity. **a**, **b** Number of (non-)overlapping barcodes in the bone marrow and extramedullary locations. Barcode analysis was restricted to the top 85% most abundant barcodes to prevent false-positive barcode calling. **c** Number of (non-)overlapping barcodes from the top 85% most abundant barcodes in the blood, pelvis and remaining body. **d**, **e** Overall barcode frequency of the top 85% most abundant barcodes that are detected or remain undetected when blood or pelvis was sampled at end-stage leukemia (one representative recipient). Statistical analysis: two-sided Mann–Whitney *U* test, *p < 0.0001.

Next, we asked whether bones with a higher leukemia cell content also contained more clones. If so, this would imply that larger locations (by chance) would be more likely to harbor relapsing clones. Similar to our previous work, cellular barcoding revealed that patient-derived leukemia clones were asymmetrically distributed across the bone marrow locations, whereas their distribution was more homogeneous across extramedullary sites (Fig. 2a, b, Supplementary Fig. 5.1A–F) [5]. Notably, in contrast to the marked variation in the leukemia cell content between small and large bones (Fig. 1b, Supplementary Fig. 2D, E), the number of barcodes varied only marginally across locations (Supplementary Fig. 5.2A–C). In larger bones (e.g. spine), barcodes were generally larger, whereas smaller bones (e.g. sternum), generally contained smaller barcodes (Supplementary Fig. 5.3A–D). Furthermore, we observed that barcodes, which were small in the overall murine xenograft, were located in a single location (Supplementary Fig. 5.3E–H), whereas larger barcodes were often present in multiple sites. Upon serial transplantation of ALL-17, the number of leukemia clones was reduced by ~40% (Supplementary Fig. 5.4A, B), and their distribution was more homogeneous compared with the primary recipients (confirmed by the Spearman rank analysis, Supplementary Fig. 5.4A, C). These data once more suggest that bone size may be an important determinant of leukemic cell content and growth.

As single-site sampling is a common practice in the clinic and in experimental studies [10–12], we assessed to what extent blood and pelvis reflect the overall barcode complexity. At sacrifice, 40–60% of the total body leukemia clones were detected in blood, whereas the remainder stayed undetected (Fig. 2c, Supplementary Fig. 5.5A–C). Detectable barcodes in the blood had an overall frequency that was significantly higher than those that remained undetected (*p* < 0.0001, Fig. 2d, Supplementary Fig. 5.6A–C). Of the undetected barcodes, 13–53% were detectable in blood samples drawn prior to sacrifice (Supplementary Fig. 5.7A–F), and 20–45% of the barcodes were never seen in blood at any measured time point. Assessment of the pelvis showed similar results (Fig. 2e, Supplementary Fig. 5.5A–C, 5.6D–F). Furthermore, combined analysis of both locations only covered 53–66% of the total body leukemia barcodes (Fig. 2c, Supplementary Fig. 5.5A–C). These data indicate that single-site sampling results in an underestimation of the clonal complexity of the disease. As each site contains unique clones (Fig. 2a, b, Supplementary Fig. 5.1A–F), one would need to sample every location to fully capture the total clonal complexity.

To summarize, we demonstrate that, at end-stage disease, murine xenografts harbor millions of human leukemia cells, with ~10% localized in blood and pelvis. Leukemia cells are derived from hundreds of leukemia-propagating cells (LPCs), which are asymmetrically distributed across skeletal sites [5, 9]. We demonstrate that, sampling of a single-site allows for half of the LPC clones to remain undetected. Therefore, multi-site sampling of xenografts will increase the yield of cells for experimental analysis and provide a more in-depth view of the clonal heterogeneity.

These observations are in apparent contrast with clonal analysis of immunoglobulin heavy chain (IgH) rearrangements in patients, which report a symmetric distribution with >80% of IgH clones present in blood and two bone marrow sites [13]. This discrepancy may be due to the choice of marker, as one IgH clone can be represented by multiple LPCs. Furthermore, the observed level of (a)symmetry may depend on the number and type of sites sampled. Although murine xenografts lack human niche factors, and may be subject to transplantation-induced clonal selection [5, 14], they provide the unique advantage of allowing sampling of nearly every cell in a given location and nearly every location in the murine body. Last, we previously showed that the degree of asymmetry depends on the number of LPCs, with more asymmetry when fewer LPCs are present [15]. This may suggest that failure to detect a relapsing clone in clinical patients may not be due to its presence below detection limits of current tests, but to its presence in a non-sampled location. Future studies in chemotherapy-treated xenografts and/or clinical patients will be needed to determine whether treatment impacts on clonal asymmetry, and whether multi-site sampling improves diagnostics, monitoring and treatment-decisions of patients with ALL.

## Supplementary information

Supplemental material
