# Correction: Quantitative distribution of patient-derived leukemia clones in murine xenografts revealed by cellular barcodes

**DOI:** 10.1038/s41375-020-0722-3

**Published:** 2020-01-31

**Authors:** Sabrina Jacobs, Albertina Ausema, Erik Zwart, Ellen Weersing, Maaike J. Kingma, Yasmine A. S. El Menshawi, Gerald de Haan, Leonid V. Bystrykh, Mirjam E. Belderbos

**Affiliations:** 1 Department of Ageing Biology and Stem Cells, European Research Institute for the Biology of Ageing (ERIBA), University Medical Center Groningen (UMCG), University of Groningen, Groningen, The Netherlands; 2 Oncode Institute and Princess Máxima Center for Pediatric Oncology, Utrecht, The Netherlands

**Keywords:** Cancer models, Acute lymphocytic leukaemia, Cancer stem cells

**Correction to: Leukemia**

10.1038/s41375-019-0695-2

This article was originally published under NPG’s License to Publish, but has now been made available under a CC BY 4.0 license. The PDF and HTML versions of the paper have been modified accordingly.

