# Supplementary material for: Quantitative distribution of patient-derived leukemia clones in murine xenografts revealed by cellular barcodes
Source: Leukemia. 2019 Dec 18;34(6):1669–74. doi: 10.1038/s41375-019-0695-2 (PMC8075919; doi:10.1038/s41375-019-0695-2)
Supplement: Supplementary file 1 — Supplemental material [file 41375_2019_695_MOESM1_ESM.pdf]

**Supplementary information**

# Quantitative distribution of patient-derived leukemia clones in murine xenografts revealed by cellular barcodes

Jacobs, S. *et al.*

## Supplementary methods

*Patient samples*

Bone marrow cells from pediatric patients with B-ALL were collected as part of routine diagnostics for suspected leukemia. Residual bone marrow cells left after clinical testing were cryopreserved for research purposes (Department of Pediatric Hematology/Oncology, University Medical Center Groningen). All patients and/or their caregivers provided a written informed consent. Procedures were approved by the Medical Ethical Committee of the University Medical Center Groningen.

In total, 5 different patient samples were selected to assess the quantitative distribution of leukemia clones across the individual locations of a murine xenograft. Based on previous study, we estimated a success rate of 50% for barcoding and engraftment. Transplantation of either non-barcoded or barcoded leukemia cells in primary recipients, followed by serial transplantation into secondary recipients will generate >15 xenografts to assess the quantitative distribution of leukemia cells in >150 individual anatomical locations.

Blinding does not apply to this study as the primary outcome measures are objective measures (cell counts, frequencies and barcodes).

*Mice*

Male Nod/SCID/IL2R$\gamma^{-/-}$ (NSG) mice, randomly assigned to an individual patient sample, were bred and housed at the Central Animal Facility of the University of Groningen. All animal experiments were approved by the Dutch Central Committee Animal Experimentation and the Animal Welfare Body of the University of Groningen.

*Barcode library construction and virus production*

An equimolar barcode library of approximately 800 barcodes, which were integrated into the pEGZ2 vector, was generated as previously described[1, 2]. Viral supernatant was produced by transfection of HEK293T cells. Hereto, HEK293T cells were cultured in a type-A gelatin-coated T75-cell culture flask in DMEM supplemented with 10% heat-inactivated FBS (Gibco, ThermoFisher Scientific), 1% penicillin

and streptomycin (Gibco, ThermoFisher Scientific) at $0.15x10^6$ cells/ml. Cells were transfected with the packaging vector pCMVΔ8.91 (3µg), the envelope vector VSV-G (0.7µg) and the barcoded pEGZ2 vector library (~800 barcodes, 3ug) in the presence of Fugene (Promega Corporation, Madison, Wisconsin, USA). Next day, culture medium was replaced by StemSpan SFEM medium (STEMCELL Technologies) supplemented with 1% penicillin and streptomycin. After approximately 24 hours, viral supernatant was collected, filtered through a 0.45µm SFCA filter and stored at $-80^0$C for later use.

*Cellular barcoding and xenotransplantation of patient-derived leukemia cells*

Patient-derived B-ALL cells were rapidly thawed. From each sample, $5x10^6$ cells were directly transplanted into 10-20 week old, sublethally irradiated NSG mice (1.0 Gy) via tail vein injection. Direct transplantation served as backup in case the first barcoding attempt failed. The remaining cells were used for lentiviral transduction with the barcoded pEGZ2 vector library. Patient sample ALL-12 was transduced using polybrene (Sigma Aldrich, Zwijndrecht, The Netherlands) according to previous procedures described by our group[1]. Patient samples ALL-15, 16, 17 and 19 were transduced using RetroNectin® as described here. Hereto, 6-well cell culture plates were pre-coated with RetroNectin® (0.05mg/ml, Takara, Kusatsu, Shiga, Japan) followed by centrifugation at 1000g, 45 min. with varying volumes of viral supernatant containing the barcoded pEGZ2 vector library (~800 barcodes). After 4 hours of incubation at $37^0$C, viral supernatant was gently removed and patient-derived leukemia cells were equally divided over the 6-well cell culture plates (2.5 – $3.5x10^6$ cells/well) followed by spinfection (900g, 45 min.). Cells were cultured in StemSpan SFEM medium (STEMCELL Technologies, Vancouver, Canada) supplemented with 10% heat-inactivated FBS (Gibco, ThermoFisher Scientific, Waltham, Massachusetts, USA), 1% penicillin and streptomycin (Gibco, ThermoFisher Scientific), human recombinant TPO (100ng/ml, PeproTech, London, UK), human recombinant IL-7 (10ng/ml, PeproTech), human recombinant FLT-3L (20ng/ml, R&D Systems, Inc., Minneapolis, Minnesota, USA) and human recombinant SCF (50ng/ml, R&D Systems). Patient samples ALL-16 and ALL-19 were successfully barcoded upon thawing (supplementary table 1). Patient sample ALL-17 required *in vivo* expansion to allow for successful barcoding. Barcoded cells were sorted for the presence of GFP using either the MoFlo XDP or Astrios flow cytometer (Beckman Coulter®, Woerden, The Netherlands) and subsequently transplanted into sublethally irradiated NSG mice with $0.5x10^6$ cells/mouse via tail vein injection. For serial transplantation, pooled bone marrow of hind legs, pelvis,

sternum and spine was used with $0.5 x 10^6$ GFP+ cells/mouse. Remaining pooled bone marrow of ALL-16 was stored at $-150^0$C for later use.

*Quantitative assessment of leukemia load*

Leukemia progression was monitored based on clinical symptoms and blood analysis every other week. When mice developed leukemia, mice were sacrificed under isoflurane and perfused with saline. Bone marrow (front and hind legs, pelvis, sternum, spine and skull) and extramedullary locations (blood, liver, spleen and brain) were collected. Single cell suspensions were prepared by crushing in erythrocyte lysis buffer followed by subsequent filtering through a 100-µm filter. The absolute number of white blood cells (WBCs) was determined by differential blood count using the Medonic CA620 Hematology analyzer (Boule Medical AB, Spanga, Sweden). The number of WBCs in peripheral blood was assessed prior to erythrocyte lysis. Hereto, a fraction of the peripheral blood was subjected to the Medonic CA620 Hematology analyzer. The output WBC count was corrected for the total circulating blood volume in an adult mouse, which is approximately 2mL. The relative frequency of leukemia cells was determined by flow cytometry using the BD FACSCanto™ II flow cytometer (BD Biosciences, San Jose, California, USA; supplementary table 2). The total leukemia cell content in any given location was calculated according to one of the formulas below:

$$\text{WBC}\ (\text{x}10^6\ \text{cells})\ \text{x}\ \text{PI}^-\ (\%\ \text{of all events})\ \text{x}\ \text{mCD45.1}^-\text{hCD45}^+(\%\ \text{of gated events});\ \text{or}$$

$$\text{WBC}\ (\text{x}10^6\ \text{cells})\ \text{x}\ \text{PI}^-\ (\%\ \text{of all events})\ \text{x}\ \text{mCD45.1}^-\text{hCD19}^+(\%\ \text{of gated events})$$

All mice were included in the analysis of the leukemia cell content in individual locations. To address the total body leukemia cell content, the leukemia cell content in all individual locations was combined. In case individual locations were not samples, an estimate was calculated based on the leukemia cell content in that specific anatomical location of the other xenografts transplanted with the same patient sample.

*Validation of the quantitative assessment of leukemia load*

The quantitative assessment of leukemia load was validated using Count Bright absolute counting beads (ThermoFisher Scientific). Bone marrow and extramedullary locations were collected and processed to single cell suspension as described before. A total of $0.5 \times 10^6$ cells per sample were stained according to supplementary table 2. The total sample volume and the input volume for flow cytometry (corresponding to $0.5 \times 10^6$ cells) were determined to correct for the total sample amount. Prior to measuring the leukemia cell content using the BD FACSCanto™ II flow cytometer, $0.26 \times 10^5$ counting beads were added to each sample. A minimum of 1000 beads were recorded. The absolute leukemia cell content was calculated according to formula below:

$$\frac{\text{Total sample volume}}{\text{Input volume}} \times \frac{\text{Total \# of added counting beads}}{\text{Recorded \# of counting beads}} \times \text{Recorded \# of mCD45.1}^{-}\text{hCD45}^{+}\text{ cells}$$

*Assessment of bone marrow compartment sizes*

Healthy, untreated male NSG mice (n=4) were sublethally irradiated and sacrificed under isoflurane anesthesia after 1-6 months. After perfusion, single-cell suspensions were prepared from the different bone marrow locations as described before. The overall compartment size was defined as the absolute number of murine WBCs present at steady-state conditions in a given anatomic location, as measured by Medonic CA620 Hematology analyzer. Alternatively, $Lin^{-}Sca\text{-}1^{+}c\text{-}Kit^{+}CD150^{+}$ (LSK-SLAM) cells (i.e. hematopoietic stem and progenitors) were used to define stem cell compartment size. The relative frequency of LSK-SLAM cells was determined by flow cytometry using the BD FACSCanto™ II flow cytometer (supplementary table 2). The total number of LSK-SLAM cells in any given bone marrow sample was calculated according to the formula below:

$$\text{WBC } (\times 10^6 \text{ cells}) \times \text{PI}^{-} \text{ (\% of all events)} \times \text{LSK-SLAM (\% of gated events)}$$

*Barcode retrieval*

Genomic DNA was isolated from cell pellets using the DNeasy Blood and Tissue (Qiagen, Hilden, Germany) or QIAamp DNA micro kit (Qiagen), according to manufacturer's instructions. Barcode sequences were amplified in a 35-cycle PCR reaction using uniquely indexed eGFP forward primers (5'-TCGGCATGGACGAGCTG-3' or 5'-GGCATGGACGAGCTGTACAAG-3') and a WPRE reverse primer (5'-GGAGAAAATGAAAGCCATACGGGAAGC-3'). Amplification of barcode sequences were

confirmed on an agarose gel. Samples that did not show a clear band on the agarose gel were subjected to a nested PCR reaction, using an extra set of outer primers (forward 5’-TGCCCGACAACCACTACCTG-‘3 and reverse 5’-AAACACAGTGCACACCACGC- ‘3). Subsequently, samples were equimolar combined in pools of max. 12 samples, which were cleaned according to the manufacturer’s instructions using the QiaQuick PCR purification kit (Qiagen). All samples were pooled together in batches that contain 200-300 different samples and the quality of the sample was confirmed using the Agilent 2100 Bioanalyzer (Agilent Technologies, Inc., Santa Clara, California, USA). Next, samples were sent for paired-end next generation sequencing on an Illumina HiSeq 2500 Platform (BaseClear, Leiden, The Netherlands).

*Barcode data processing*

Raw sequencing reads with a minimal Phred quality score of 30 were collapsed (github: https://github.com/erikzwart/collapse-multiplex-barcodes). Collapsed reads were processed by performing an exact match of the sample tag sequence. Next, barcodes with either the GAANNNACNNNGTNNNCGNNNTANNNCANNNTAAGGAC or AGGNNNACNNNGTNNNCGNNNTANNNCANNNTGNNNGAC backbone were retrieved using Motif Occurrence Detection Suite (MOODS, 1.0.2 https://github.com/jhkorhonen/MOODS/wiki/Installation-and-usage-(MOODS-1.0)). The generated list contained all possible barcodes per sample, which was further filtered by applying a Hamming distance of one to delete barcodes that differ one base. Samples that belonged to the same experiment were pooled into one table.

Data were further processed and analyzed using custom R scripts. First, we calculated the theoretic chance of having multiple barcode integrations into a single cell (supplementary table 2), and subsequently analyzed the actual number of cells having multiple barcode integrations. This type of analysis is based on barcode patterns: Two distinct barcodes that follow the exact same distribution pattern over time, or across the anatomical locations in all recipients, are likely the consequence of two barcode integrations into a single cell. Hereto, we performed linear regression analysis, where the frequency of each individual barcode across all anatomical locations of every recipient was compared to that of all other barcodes. In case the slope and $R^2$ value of two distinct barcodes were close to one,

defined as *slope – 1 < 0.05* and the $R^2 > 0.99$, these barcodes were defined as multiple integrations into a single cell and one of the barcodes was removed from further data analysis. Second, additional filtering steps were applied to remove low-noise barcodes. Hereto, the degree of noise was first visualized in a histogram plot. This plot shows a biphasic distribution, where the peak centered at the very low read frequencies likely represents sequencing noise. To select true barcode clones, we selected barcodes that were (i) present in more than three samples, and (ii) had an overall read frequency of ≥0.5‰. To confirm removal of low-noise barcodes, we re-plotted the barcode frequencies in a histogram. The resulting barcode frequency table was used as input file for further data analysis. Barcode analysis was restricted to ALL-17 and ALL-19, because the primary recipients of ALL-16 consisted of five barcodes, which was reduced to two barcodes in the secondary recipients. To prevent further false-positive barcode calling from deep sequencing data[2], we restricted our analysis to the top 85% most abundant barcodes. Raw barcode data are available upon request.

*Definitions of clonality and barcode library size*

Here, we defined clonality as the number of distinct barcodes in a population, and used a library consisting of ~800 individually prepared barcode preps to study the number of clones and their dynamics. Using a library of known content and complexity has the advantage of facilitating signal-noise discrimination, thus allowing for more accurate barcode calling. However, in this manuscript, the size of our library was far smaller than the number of cells subjected to the barcoding procedure. Nonetheless, we are confident that the 800-barcode library is of sufficient size for our experimental aims, because multiple observations indicate that its size exceeds the number of *clonogenic* (i.e. leukemia-propagating) cells in our *in vivo* experiments by several-fold:

First, in most *in vivo* experiments, we did not capture the full library content, which suggests that not all barcodes are integrated into a clone-producing cell. In contrast, in *in vitro* experiments with barcoded cell lines, of which every cell is in theory self-renewing, we repeatedly recovered the full library. The fact that we did not capture the full library from the leukemia xenografts, indicates an abundance of barcodes compared to the number of leukemia cells that produce a detectable clone in our model.

Second, similar to our previous publications, we observed a drastic reduction in the number of barcodes upon serial transplantation[1, 3]. We suspect that the xenotransplantation procedure itself creates a selective bottleneck. In this previous work[1] We performed random model calculations to

calculate the estimate the mortality rate of the transplanted cell population, and demonstrate that >90% of administered cells fail to engraft. This was a key observation to reassess the clonal barcode representation in our survived cell population *in vivo*. With model analysis (implemented as custom python script), we concluded that at least 80% of the surviving barcoded clones in our experiments represent a single-cell origin.

The model analysis works as follows:

1. Set a population of *x* cells (e.g. 1000).
2. Randomly label the cells within this population with a barcode library of size *y* (e.g. 10).
3. Check the barcode representation of the population and perform statistics of redundant barcoding.
4. Set the population size and a specific barcode size *z*. Hereto, select a random fraction of all barcoded cells until a certain number of barcodes is reached. This will address the question about the size of the subset to reduce the barcode library to the needed fraction.
5. Adjust the parameters to the actual experimental parameters: *x* equals number of xenotransplanted cells; *y* equals the barcode library size; *z* equals barcode numbers retrieved upon transplantation.
6. Calculate frequency of barcodes that represents a single-cell origin and residual redundancy of barcoding.

Third, the observed level of anatomic asymmetry in barcode distribution supports the conclusion that the great majority, if not each, of the barcodes represent a single LPC. Similar to our explanation above, if each barcode would represent multiple LPCs, any functional difference would be averaged out and barcode distribution would be symmetric. An extreme example of this is GFP: Because the population of GFP-positive and GFP-negative cells each consist of multiple LPCs, their distribution across anatomic locations is highly symmetric. In contrast, the asymmetric distribution of barcode clones indicates that most barcodes represent single LPCs.

*Statistical analysis*

Statistical analysis and data visualization was performed in R (version 3.5.3) using packages “eulerr” and “ggplot2”[4–6]. Data significance was routinely tested using standard functions in R. Differences

between groups were assessed by two-sided Mann Whitney U and correlations by Spearman rank analysis. The number of barcodes within a sample was expressed using the Shannon diversity index and derivatives, as previously described[2]. The quartile coefficient of dispersion (QCD) was used to express the degree of variation for a variable between recipients. Graphpad Prism 5.0 was used to perform a nonlinear regression analysis (least squares fit, forced intercept of 0 and <5% outlier detection) between the single and multiple platform method used to quantify absolute leukemia cell content. Data is expressed either as mean ± standard deviation or median ± interquartile range (IQR). Statistical significance was defined as $p<0.05$.

## Supplementary figures

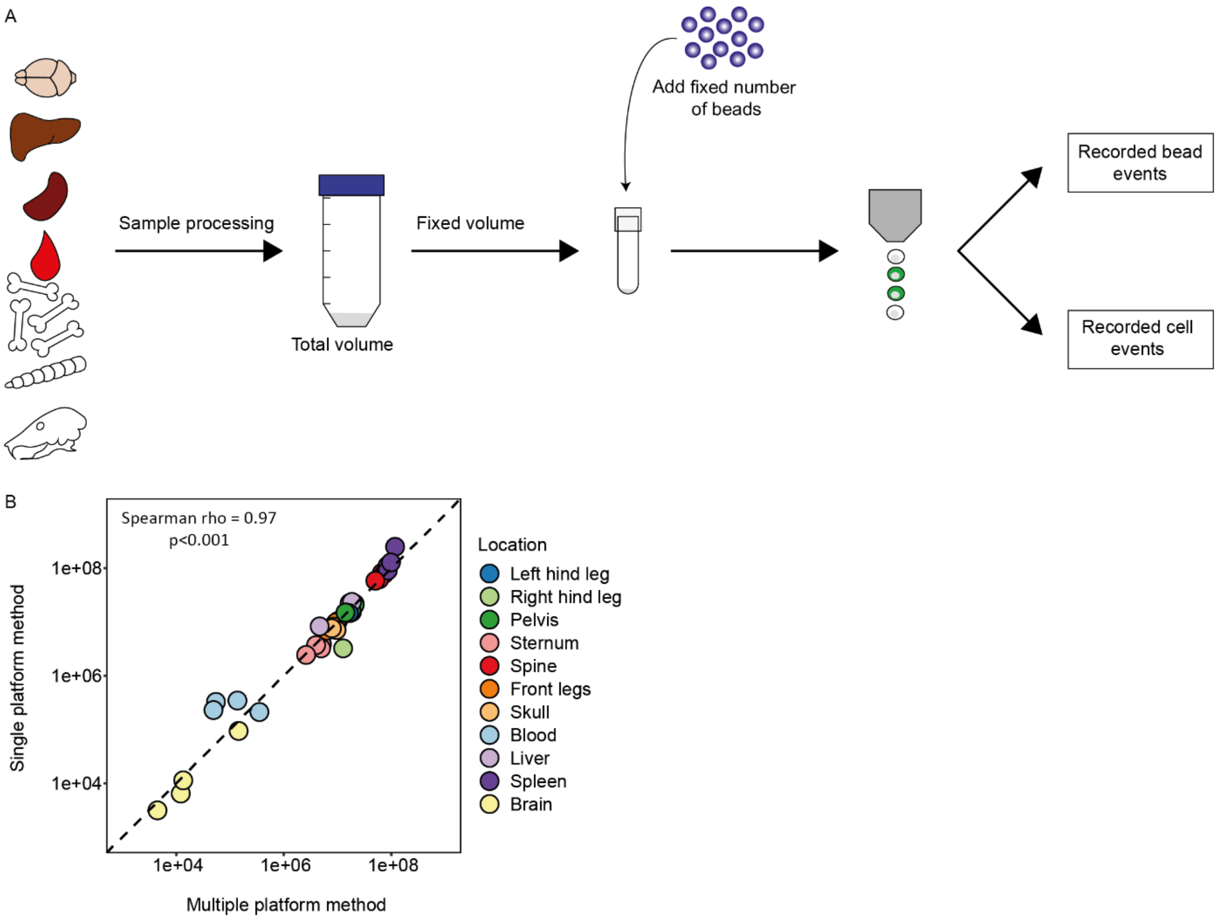

*Supplementary figure 1: Validation of a multiple platform method to quantify the leukemia cell content*

(A) Experimental design to quantify the leukemia cell content using a single platform method, which is based on calibrated beads by flow cytometry. (B) Spearman rank correlation of 0.97 between a single and multiple platform method to quantify leukemia cell content (n=4). Individual locations are plotted along the perfect line of y=x (dotted line). Most values are close to the expected y=x (nonlinear regression, slope=0.94±0.01, $R^2$=0.99). For subsequent experiments, the multiple platform method was used.

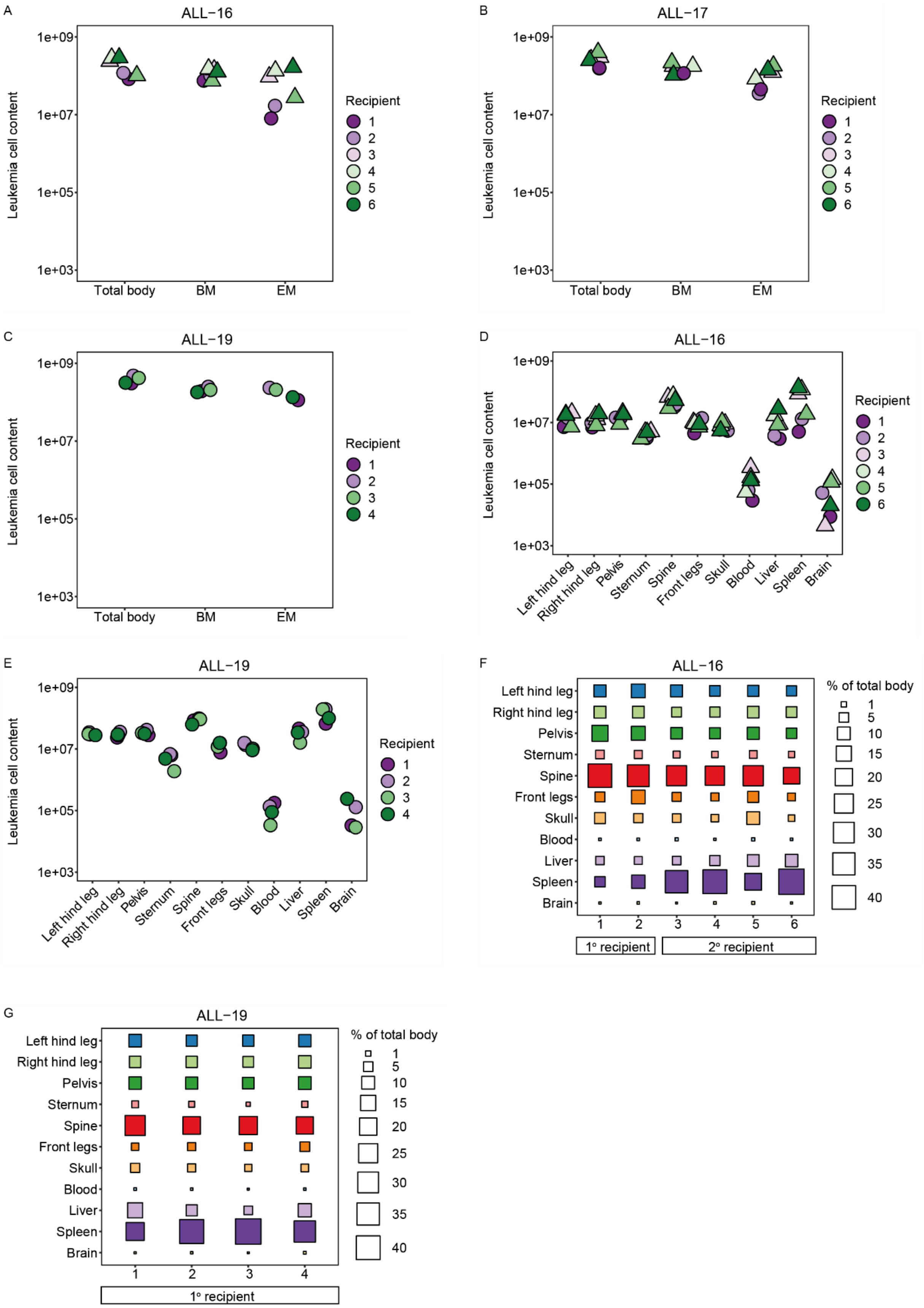
A
ALL−16
Leukemia cell content
1e+09
1e+07
1e+05
1e+03
Total body
BM
EM
Recipient
1
2
3
4
5
6
B
ALL−17
Leukemia cell content
1e+09
1e+07
1e+05
1e+03
Total body
BM
EM
Recipient
1
2
3
4
5
6
C
ALL−19
Leukemia cell content
1e+09
1e+07
1e+05
1e+03
Total body
BM
EM
Recipient
1
2
3
4
D
ALL−16
Leukemia cell content
1e+09
1e+07
1e+05
1e+03
Left hind leg
Right hind leg
Pelvis
Sternum
Spine
Front legs
Skull
Blood
Liver
Spleen
Brain
Recipient
1
2
3
4
5
6
E
ALL−19
Leukemia cell content
1e+09
1e+07
1e+05
1e+03
Left hind leg
Right hind leg
Pelvis
Sternum
Spine
Front legs
Skull
Blood
Liver
Spleen
Brain
Recipient
1
2
3
4
F
ALL−16
Left hind leg
Right hind leg
Pelvis
Sternum
Spine
Front legs
Skull
Blood
Liver
Spleen
Brain
1
2
3
4
5
6
1° recipient
2° recipient
% of total body
1
5
10
15
20
25
30
35
40
G
ALL−19
Left hind leg
Right hind leg
Pelvis
Sternum
Spine
Front legs
Skull
Blood
Liver
Spleen
Brain
1
2
3
4
1° recipient
% of total body
1
5
10
15
20
25
30
35
40

*Supplementary figure 2: Quantitative distribution of leukemia cell content in murine xenografts*

(A-C) The leukemia cell content in the total body, bone marrow (hind and front legs, pelvis, sternum, spine and skull) and extramedullary (blood, liver, spleen and brain) sites of murine xenografts. Symbols refer to primary (circle) and secondary (triangle) recipients. (D-E) The leukemia cell content in the individual locations of murine xenografts. Symbols refer to primary (circle) and secondary (triangle) recipients. (F-G) Relative contribution of each individual location to the total body leukemia cell content of murine xenografts.

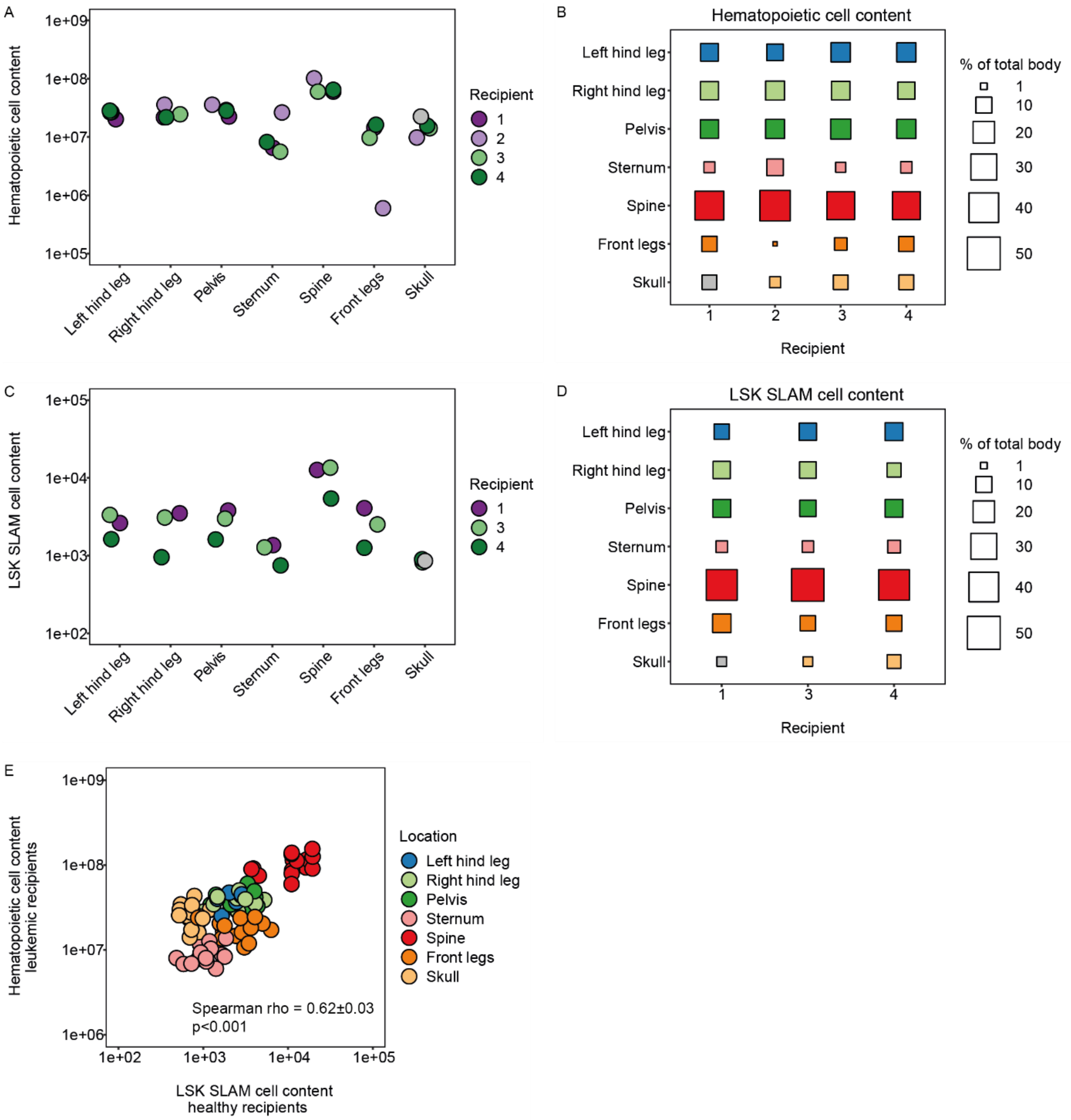

A
Hematopoietic cell content
1e+09
1e+08
1e+07
1e+06
1e+05
Left hind leg
Right hind leg
Pelvis
Sternum
Spine
Front legs
Skull
Recipient
1
2
3
4
B
Hematopoietic cell content
% of total body
1
10
20
30
40
50
Recipient
C
LSK SLAM cell content
1e+05
1e+04
1e+03
1e+02
D
LSK SLAM cell content
E
Hematopoietic cell content leukemic recipients
LSK SLAM cell content healthy recipients
Location
Spearman rho = 0.62±0.03
p<0.001

*Supplementary figure 3: Leukemia load is proportional to the compartment size*

(A, C) The absolute number of hematopoietic cells and LSK SLAM hematopoietic stem and progenitor cells in the individual bone marrow location of non-paired healthy NSG mice. (B, D) The contribution of each individual bone marrow location to the total hematopoietic cell content or the total LSK SLAM cell content in the bone marrow of non-paired healthy NSG mice. Grey square; skull of recipient 1 was not sampled and is the average of recipients 2-4. (E) The correlation between the number of hematopoietic cells in the bone marrow of leukemic (n=16) and the number of LSK SLAM cells in healthy (n=4) NSG mice. Hereto, we used a random-comparison model which randomly assigned one out of the four healthy mice to one out of the sixteen leukemic mice to calculate the Spearman rank correlation (n=1000 random comparisons). Data expressed as mean ± SD.

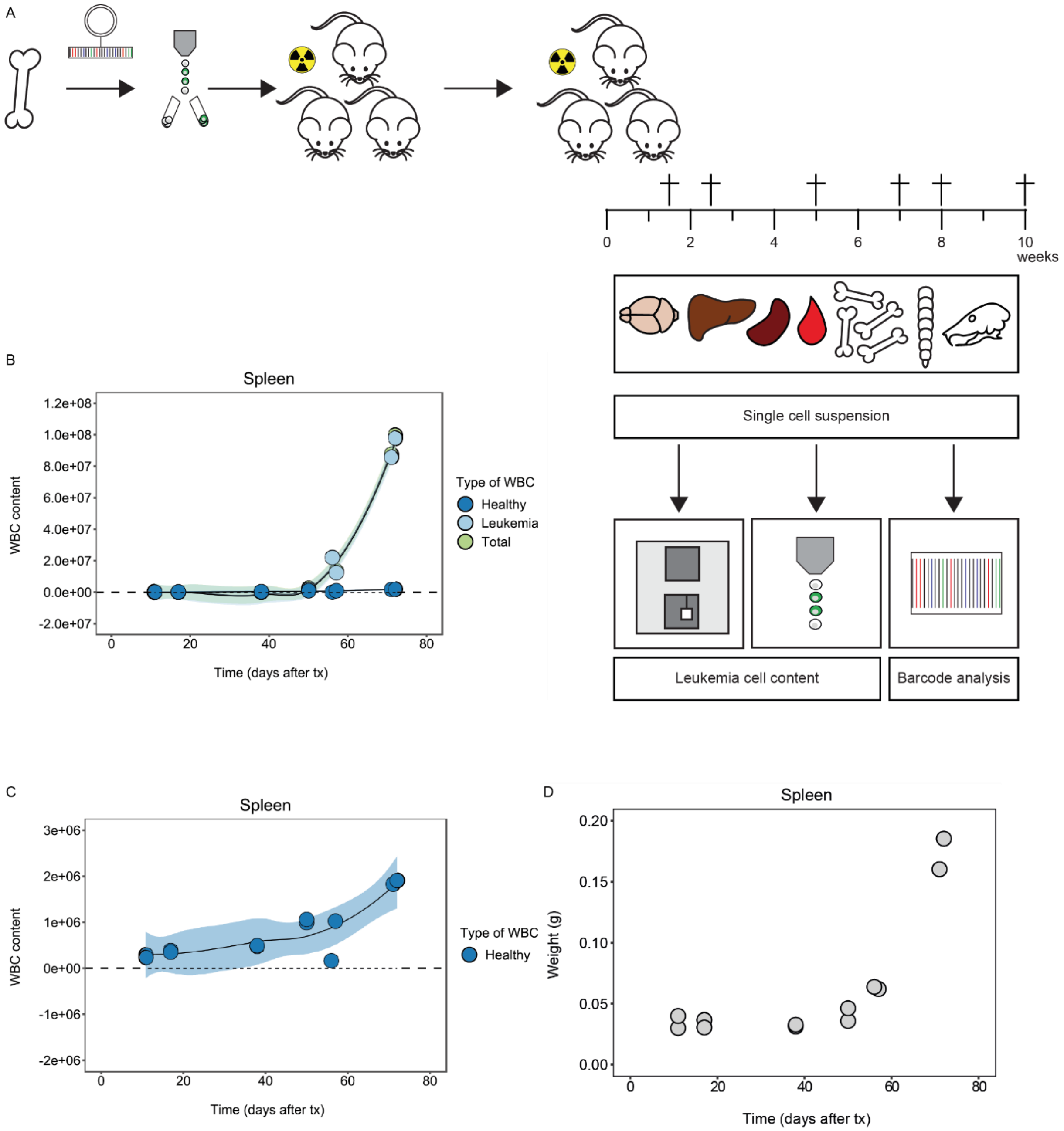
A
0
2
4
6
8
10
weeks
Single cell suspension
Leukemia cell content
Barcode analysis
B
Spleen
WBC content
1.2e+08
1.0e+08
8.0e+07
6.0e+07
4.0e+07
2.0e+07
0.0e+00
-2.0e+07
0
20
40
60
80
Time (days after tx)
Type of WBC
Healthy
Leukemia
Total
C
Spleen
WBC content
3e+06
2e+06
1e+06
0e+00
-1e+06
-2e+06
0
20
40
60
80
Time (days after tx)
Type of WBC
Healthy
D
Spleen
Weight (g)
0.20
0.15
0.10
0.05
0.00
0
20
40
60
80
Time (days after tx)

*Supplementary figure 4.1. Leukemia cell content during different stages of disease progression*

(A) Experimental design to quantify the leukemia cell content during different stages of disease progression. Patient-derived bone marrow cells of ALL-16 were barcoded, sorted for GFP and transplanted into sublethally irradiated NSG mice. Mice were sacrificed during different stages during disease progression (n=2 per time point). Individual locations were analyzed for their leukemia cell content and barcode composition. (B) The absolute number of WBCs in the spleen of leukemic mice during disease progression. Distinctions were made between leukemic cells (light blue), healthy murine WBCs (dark blue) and the total number of WBCs (light green). Every dot represents a mouse. Smoothing method 'loess' with confidence interval set at 95%. (C) Number of healthy murine WBCs in spleen of leukemic mice during disease progression. (D) Spleen weight of leukemic mice during disease progression.

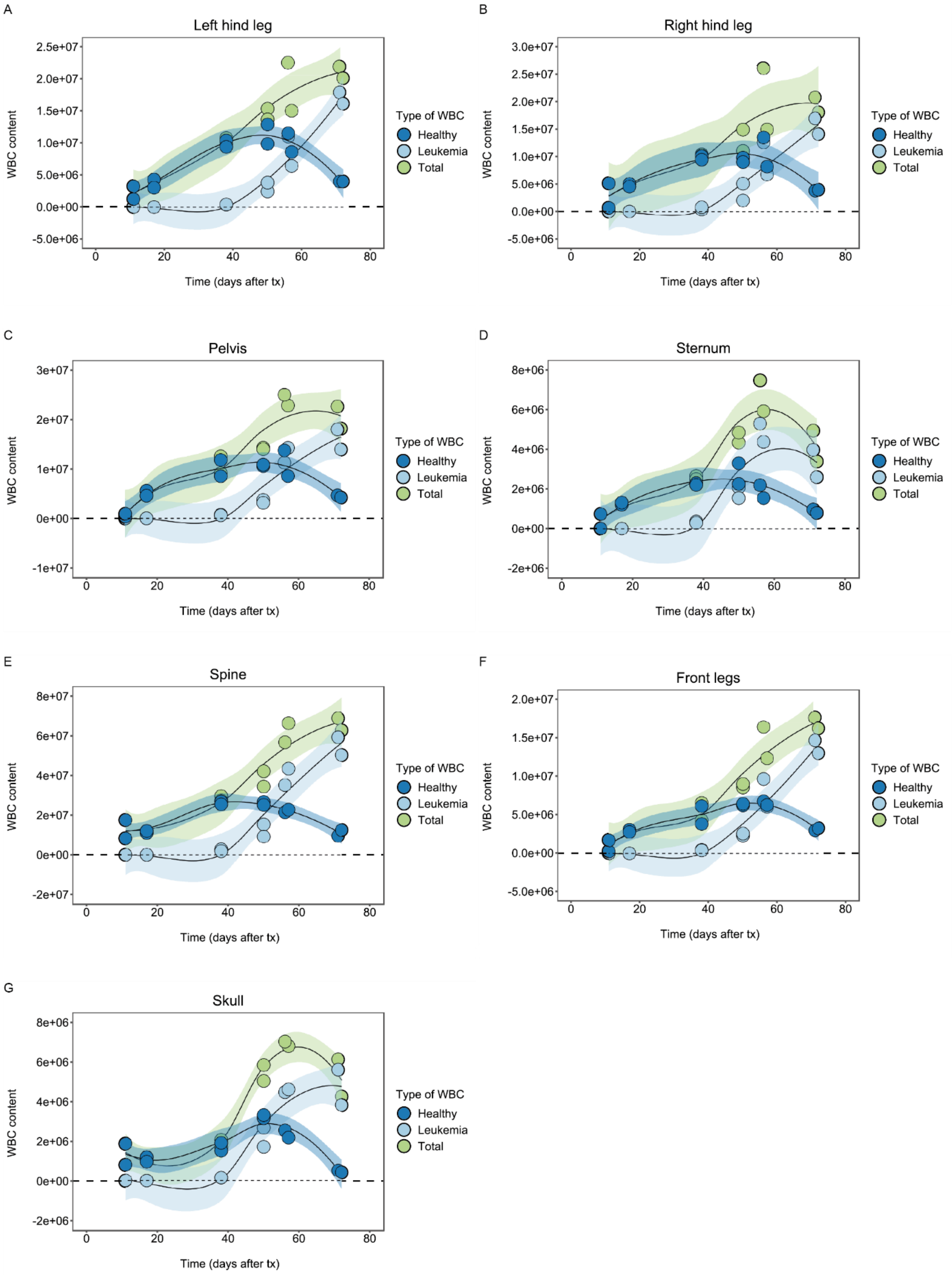
A
Left hind leg
B
Right hind leg
C
Pelvis
D
Sternum
E
Spine
F
Front legs
G
Skull
WBC content
Time (days after tx)
Type of WBC
Healthy
Leukemia
Total

*Supplementary figure 4.2. Leukemia cell content during different stages of disease progression*

(A-G) The absolute number of WBCs in the individual bone marrow locations of leukemic mice during different stages of disease progression. Distinctions were made between leukemic cells (light blue), healthy murine WBCs (dark blue) and the total number of WBCs (light green). Every dot represents a mouse. Smoothing method ‘loess’ with confidence interval set at 95%.

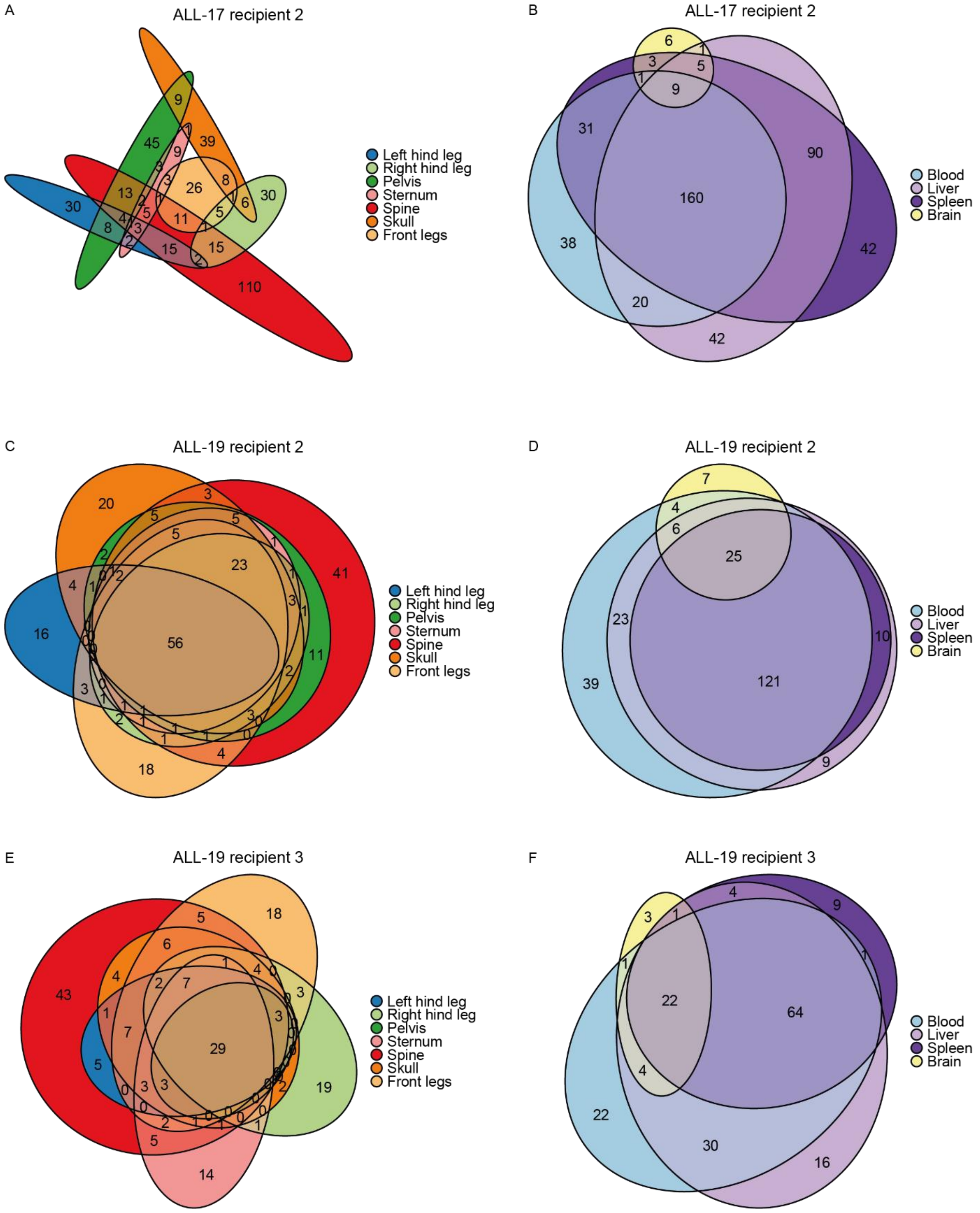
A
ALL-17 recipient 2
Left hind leg
Right hind leg
Pelvis
Sternum
Spine
Skull
Front legs
B
ALL-17 recipient 2
Blood
Liver
Spleen
Brain
C
ALL-19 recipient 2
Left hind leg
Right hind leg
Pelvis
Sternum
Spine
Skull
Front legs
D
ALL-19 recipient 2
Blood
Liver
Spleen
Brain
E
ALL-19 recipient 3
Left hind leg
Right hind leg
Pelvis
Sternum
Spine
Skull
Front legs
F
ALL-19 recipient 3
Blood
Liver
Spleen
Brain

*Supplementary figure 5.1. Asymmetrical distribution of human leukemia clones in the murine bone marrow*

(A-F) Number of (non-)overlapping barcodes from the top 85% most abundant barcodes in the bone marrow and extramedullary locations of murine xenografts.

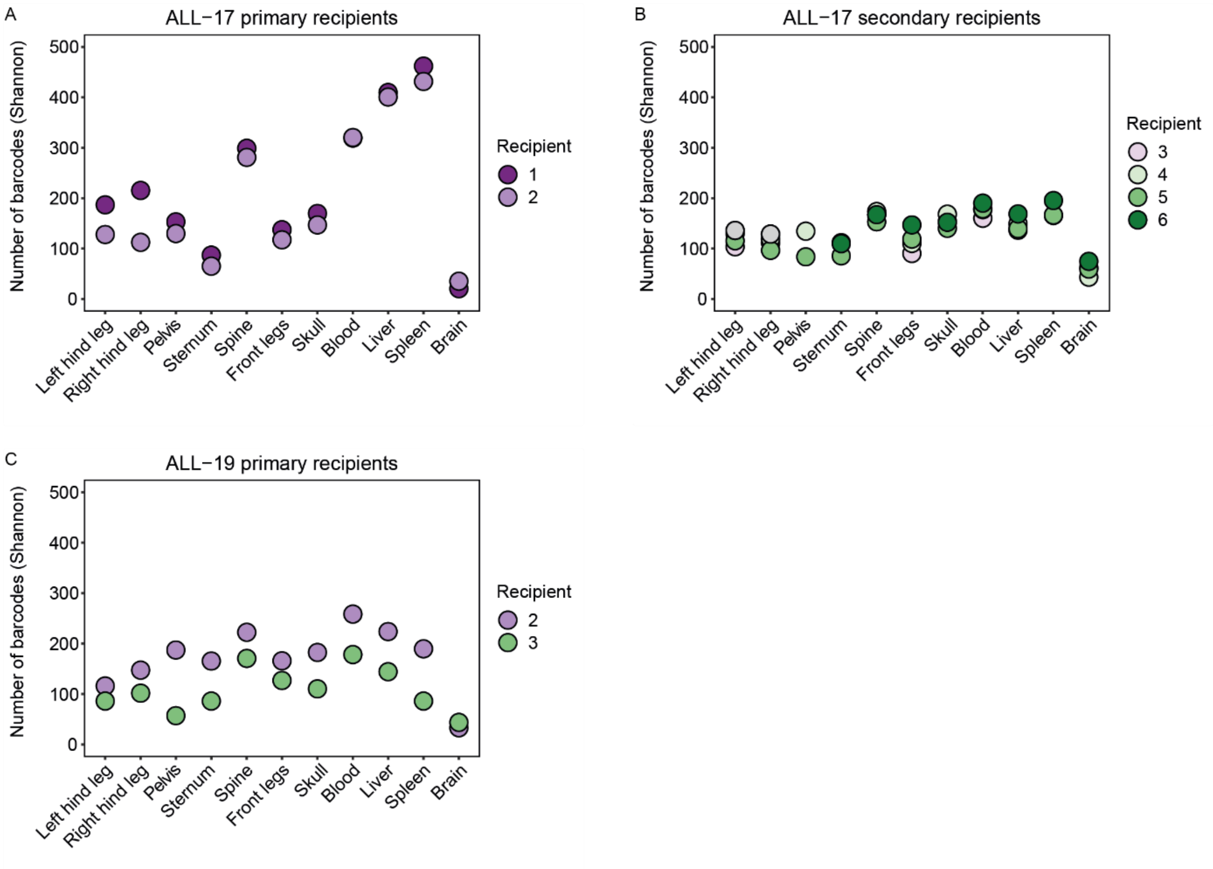
A
ALL−17 primary recipients
Number of barcodes (Shannon)
500
400
300
200
100
0
Left hind leg
Right hind leg
Pelvis
Sternum
Spine
Front legs
Skull
Blood
Liver
Spleen
Brain
Recipient
1
2
B
ALL−17 secondary recipients
Recipient
3
4
5
6
C
ALL−19 primary recipients
Recipient
2
3

*Supplementary figure 5.2. Barcode sizes in individual anatomical locations of murine xenografts*

(A-C) The number of barcodes – expressed as Shannon count – in the individual anatomical locations.

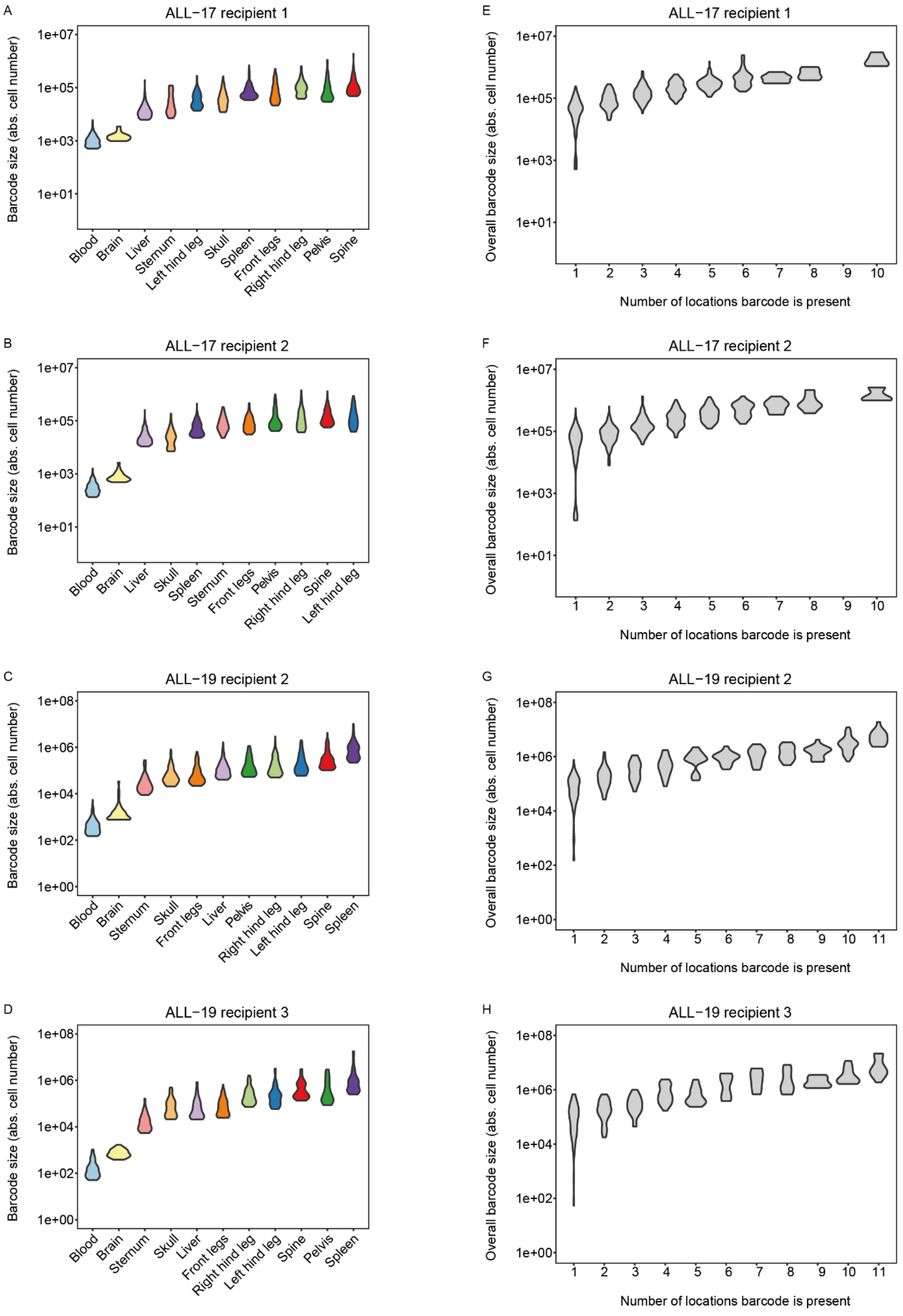
A
ALL−17 recipient 1
Barcode size (abs. cell number)
1e+07
1e+05
1e+03
1e+01
Blood
Brain
Liver
Sternum
Left hind leg
Skull
Spleen
Front legs
Right hind leg
Pelvis
Spine
E
ALL−17 recipient 1
Overall barcode size (abs. cell number)
1e+07
1e+05
1e+03
1e+01
1
2
3
4
5
6
7
8
9
10
Number of locations barcode is present
B
ALL−17 recipient 2
Barcode size (abs. cell number)
1e+07
1e+05
1e+03
1e+01
Blood
Brain
Liver
Skull
Spleen
Sternum
Front legs
Pelvis
Right hind leg
Spine
Left hind leg
F
ALL−17 recipient 2
Overall barcode size (abs. cell number)
1e+07
1e+05
1e+03
1e+01
1
2
3
4
5
6
7
8
9
10
Number of locations barcode is present
C
ALL−19 recipient 2
Barcode size (abs. cell number)
1e+08
1e+06
1e+04
1e+02
1e+00
Blood
Brain
Sternum
Skull
Front legs
Liver
Pelvis
Right hind leg
Left hind leg
Spine
Spleen
G
ALL−19 recipient 2
Overall barcode size (abs. cell number)
1e+08
1e+06
1e+04
1e+02
1e+00
1
2
3
4
5
6
7
8
9
10
11
Number of locations barcode is present
D
ALL−19 recipient 3
Barcode size (abs. cell number)
1e+08
1e+06
1e+04
1e+02
1e+00
Blood
Brain
Sternum
Skull
Liver
Front legs
Right hind leg
Left hind leg
Spine
Pelvis
Spleen
H
ALL−19 recipient 3
Overall barcode size (abs. cell number)
1e+08
1e+06
1e+04
1e+02
1e+00
1
2
3
4
5
6
7
8
9
10
11
Number of locations barcode is present

*Supplementary figure 5.3. Barcode sizes in individual anatomical locations of murine xenografts*

(A-D) Barcode size – expressed as absolute cell number – of the top 85% most abundant barcodes in each individual anatomical location. (E-H) Overall barcode size of the top 85% most abundant barcodes – expressed as absolute cell number – per number of locations in which the barcode was present.

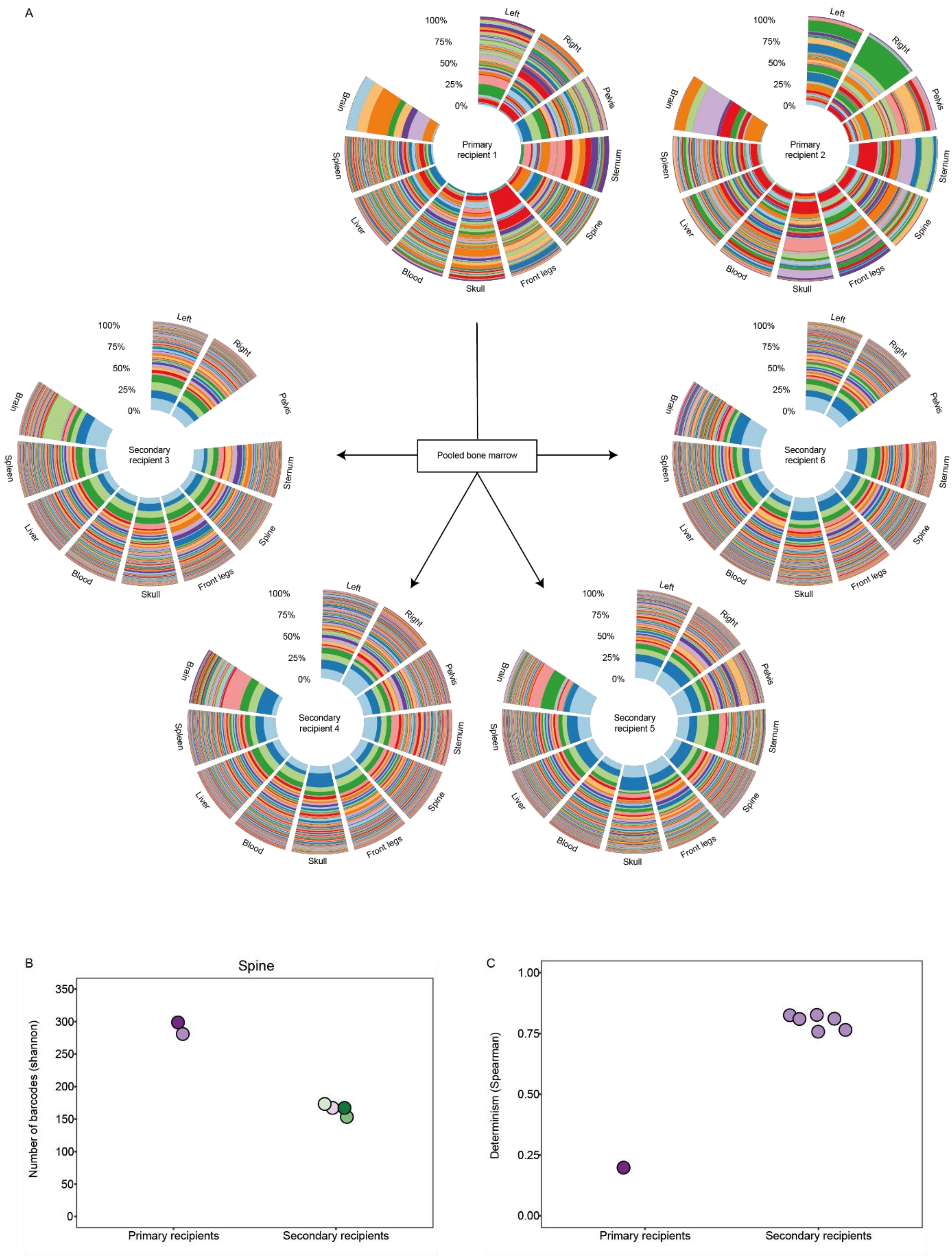
A
Primary recipient 1
Primary recipient 2
Secondary recipient 3
Secondary recipient 4
Secondary recipient 5
Secondary recipient 6
Pooled bone marrow
100%
75%
50%
25%
0%
Left
Right
Pelvis
Sternum
Spine
Front legs
Skull
Blood
Liver
Spleen
Brain
B
Spine
Number of barcodes (shannon)
350
300
250
200
150
100
50
0
Primary recipients
Secondary recipients
C
Determinism (Spearman)
1.00
0.75
0.50
0.25
0.00
Primary recipients
Secondary recipients

*Supplementary figure 5.4. Clonal selection upon serial transplantation of ALL-17*

(A) Top 100 most abundant barcodes in individual locations of serially transplanted xenografts. Each circle represents one mouse, every segment within a circle represent an anatomical location and every color represents one barcode. For serial transplantation into recipients 3-6, pooled bone marrow of the left hind leg ('Left'), right hind leg ('Right'), pelvis, sternum and spine derived from recipient 1 was used. (B) The number of barcodes – expressed as Shannon count – in the spine bone marrow of primary recipients 1 and 2, and secondary recipients 3-6. (C) Determinism (predictability) of barcode composition in primary and secondary recipients. Hereto, we compared the Spearman rank coefficient for the top 85% barcode composition in the spine bone marrow between the two primary recipients, and the four secondary recipients.

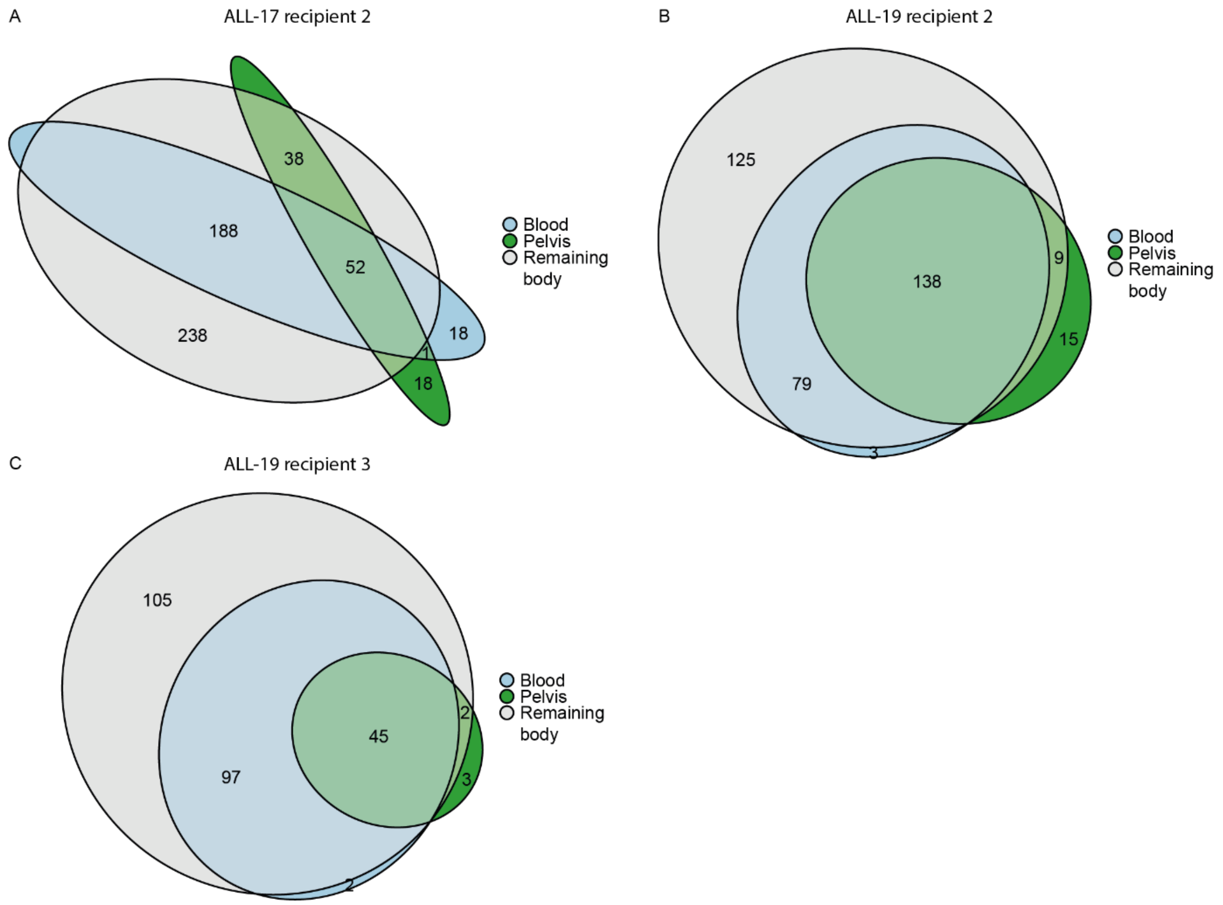
A
ALL-17 recipient 2
38
188
52
238
18
1
18
Blood
Pelvis
Remaining body
B
ALL-19 recipient 2
125
9
138
15
79
3
Blood
Pelvis
Remaining body
C
ALL-19 recipient 3
105
2
45
97
3
2
Blood
Pelvis
Remaining body

*Supplementary figure 5.5: Underestimation of clonal complexity by single-site sampling*

(A-C) Number of (non-)overlapping barcodes from the top 85% most abundant barcodes in the blood, pelvis and the remaining body.

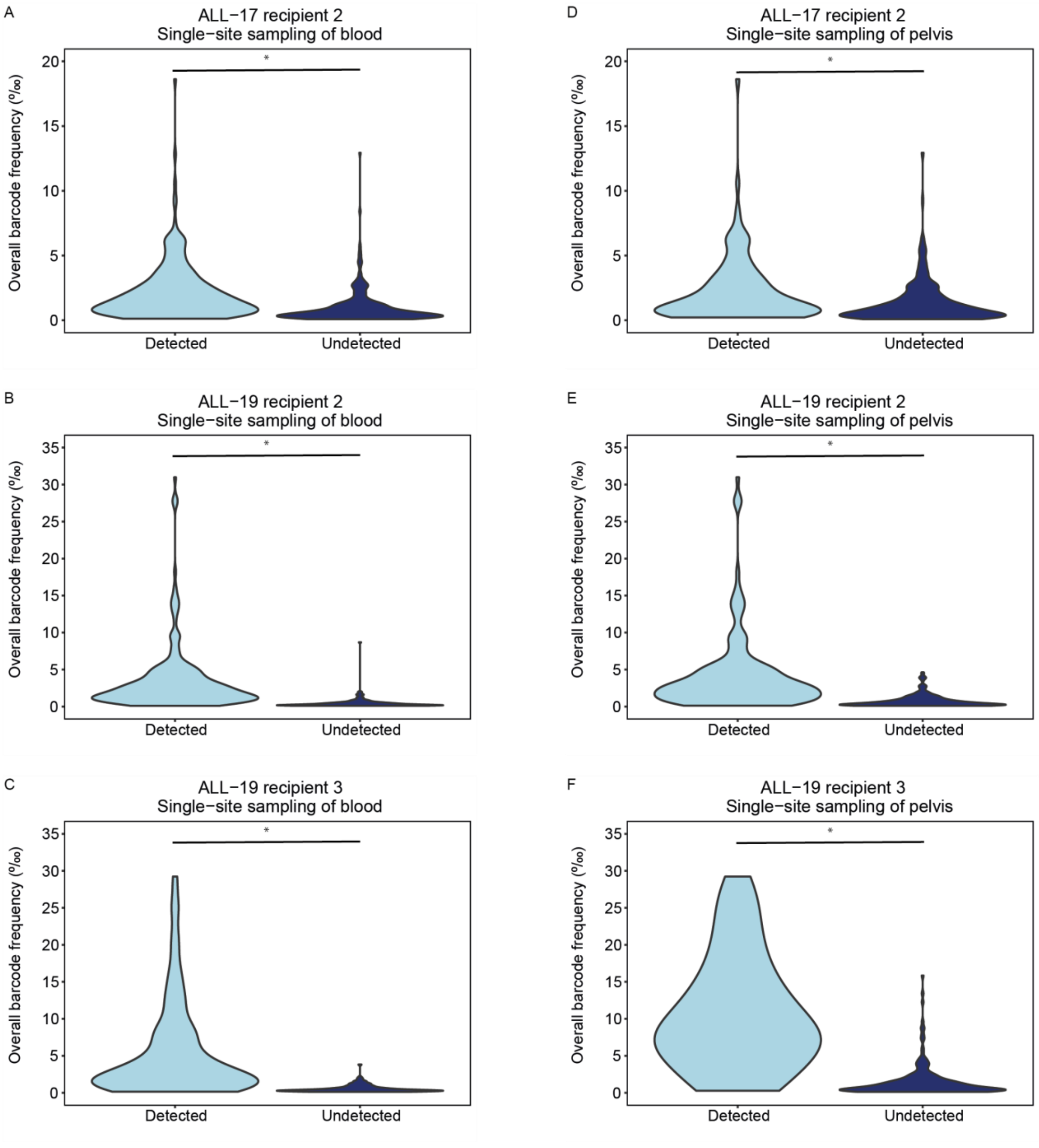
A
ALL−17 recipient 2
Single−site sampling of blood
D
ALL−17 recipient 2
Single−site sampling of pelvis
B
ALL−19 recipient 2
Single−site sampling of blood
E
ALL−19 recipient 2
Single−site sampling of pelvis
C
ALL−19 recipient 3
Single−site sampling of blood
F
ALL−19 recipient 3
Single−site sampling of pelvis
Overall barcode frequency (‰)
*
0
5
10
15
20
25
30
35
Detected
Undetected

*Supplementary figure 5.6. Underestimation of clonal complexity by single-site sampling*

(A-F) Overall barcode frequency of the top 85% most abundant barcodes that are detected or remain undetected when blood or pelvis was sampled at end-stage leukemia. Statistical analysis: two-sided Mann-Whitney U test, * $p<0.0001$.

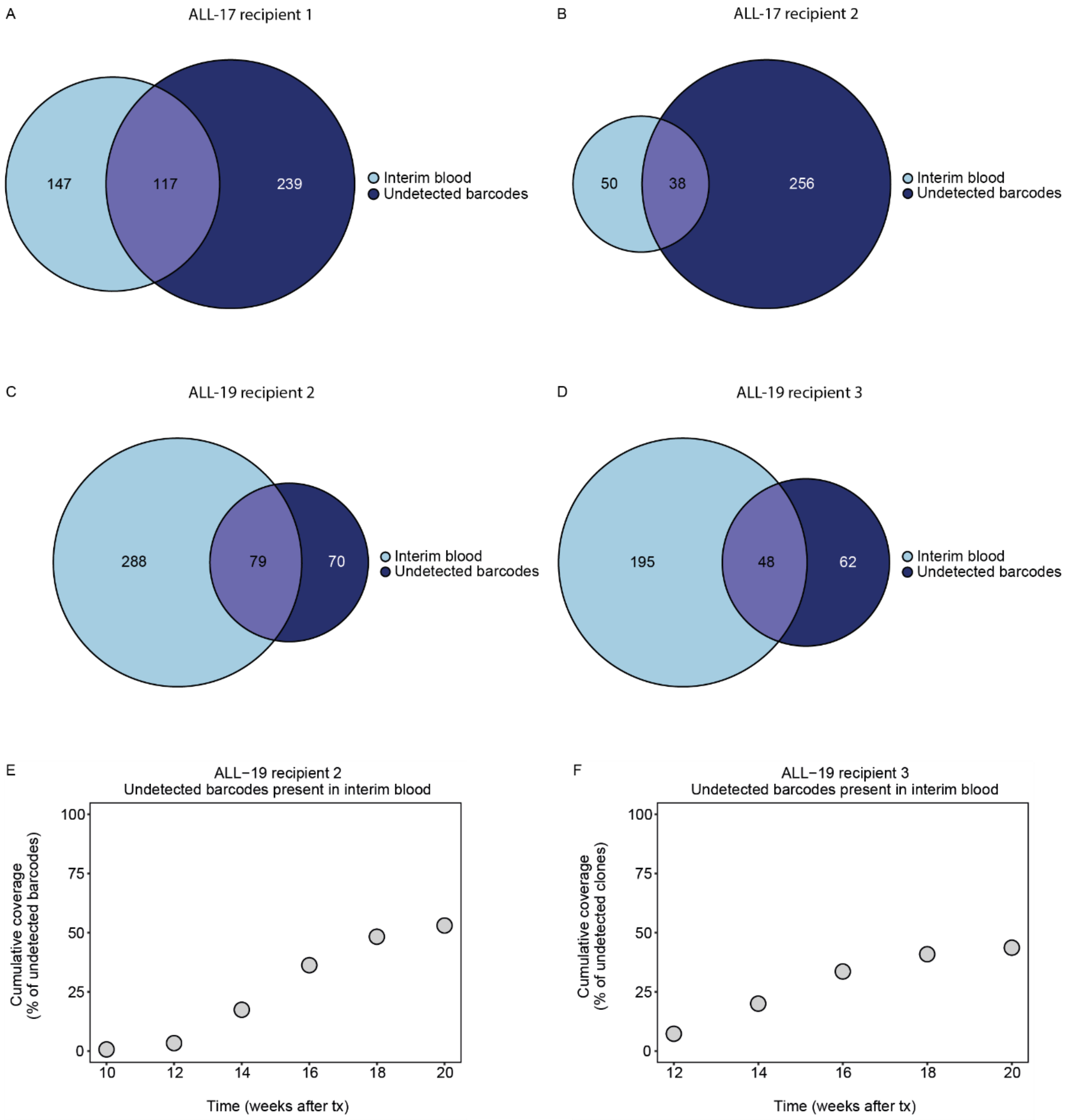
A
ALL-17 recipient 1
147
117
239
Interim blood
Undetected barcodes
B
ALL-17 recipient 2
50
38
256
Interim blood
Undetected barcodes
C
ALL-19 recipient 2
288
79
70
Interim blood
Undetected barcodes
D
ALL-19 recipient 3
195
48
62
Interim blood
Undetected barcodes
E
ALL−19 recipient 2
Undetected barcodes present in interim blood
Cumulative coverage (% of undetected barcodes)
100
75
50
25
0
10
12
14
16
18
20
Time (weeks after tx)
F
ALL−19 recipient 3
Undetected barcodes present in interim blood
Cumulative coverage (% of undetected clones)
100
75
50
25
0
12
14
16
18
20
Time (weeks after tx)

*Supplementary figure 5.7. Underestimation of clonal complexity by single-site sampling*

(A-D) The number of undetected clones that are detectable in interim blood samples when single-site sampling of the blood, at end-stage leukemia, is performed. (E-F) The cumulative coverage of undetected clones for single-site sampling of the blood at end-stage leukemia by multiple sampling of interim blood samples.

**Supplementary tables**

*Supplementary table 1*

Characteristics of patient samples used for transplantation. Abbreviations: multiplicity of infection (MOI), transduction efficiency (TE).

| Patient | Age at Dx (y) | WBC count ($10^9$/L) | Blasts (%) | Karyotype | Cytogenetic aberrations | Engraftment | TE (%) | Predicted MOI (%) | Cells with multiple integrations (double hits) | Barcoding | Barcoded recipients | Un-barcoded recipients |
|---|---|---|---|---|---|---|---|---|---|---|---|---|
| **ALL-12** | 10 | 27 | 76 | 46, XY, der(4)t(1;4)(q25; p16)[3] | | No | 0.19 | n.a. | n.a. | n.a. | 2 | 2 |
| **ALL-15** | 12 | 123 | 94 | 46, XX[30] | del1-5 PAX5 | No | 0.04 | n.a. | n.a. | n.a. | 1 | 1 |
| **ALL-16** | 10 | 57 | 97 | 47, XX, +X, -14, +mar[6]/47, idem, del(9)(p21)[9]/46, idem, der(5)t(5;9)(q3?3;q12), -9[2]/46, XX[5] | amp5 PAX5; delCDKN2A/B; gain CRLF2 | Yes | 2.61 | n.a. | n.a. | monoclonal | 17 | 1 |
| **ALL-17** | 18 | 295 | 79 | 46, XY | BCR-ABL | Yes | 9.00 | 0.38 | 2 | polyclonal | 6 | 0 |
| **ALL-19** | 6 | 7.6 | 45 | 54,XY,+X,+Y,+8, +9,+14,+18,+21, +21[11]/46,XY[4] | DelIKZF1 | Yes | 7.50 | 0.27 | 1 | polyclonal | 2 | 2 |

*Supplementary table 2*

Antibodies used for flow cytometry to determine leukemia or LSK SLAM frequency.

| **Antibody** | **Clone** | **Fluorochrome** | **Company** | **Cat. #** |
|---|---|---|---|---|
| *Human leukemia* | | | | |
| Anti-human CD19 | HIB19 | PE | Biolegend | 302208 |
| Anti-human CD45 | HI30 | PE/Cy7 | Biolegend | 304016 |
| Anti-human CD34 | 8G12 | APC | BD Biosciences | 345604 |
| Anti-mouse CD45.1 | A20 | Pacific blue | Biolegend | 110722 |
| *Mouse LSK-SLAM* | | | | |
| Anti-mouse Ly-6A/E (Sca-1) | D7 | Pacific blue | Biolegend | 108120 |
| Anti-mouse CD117 (c-Kit) | 2B8 | PE | Biolegend | 105808 |
| Anti-mouse CD150 (SLAM) | TC15-12F12.2 | PE/Cy7 | Biolegend | 115914 |
| Anti-mouse CD48 | HM48-1 | Alexa Fluor® 647 | Biolegend | 103416 |
| Anti-mouse/human CD45R/B220 | RA4-6B2 | Alexa Fluor® 700 | Biolegend | 103232 |
| Anti-mouse/human CD11b | M1/70 | Alexa Fluor® 700 | Biolegend | 101222 |
| Anti-mouse CD3 | 17A2 | Alexa Fluor® 700 | Biolegend | 100216 |
| Anti-mouse Ly-6G/Ly-6C (Gr-1) | RB6-8C5 | Alexa Fluor® 700 | Biolegend | 108422 |
| Anti-mouse TER-119/Erythroid Cells Antibody | TER-119 | Alexa Fluor® 700 | Biolegend | 116220 |

**Supplementary references**

1. Belderbos ME, Koster T, Ausema B, et al (2017) Clonal selection and asymmetric distribution of human leukemia in murine xenografts revealed by cellular barcoding. Blood 129:3210 LP – 3220. https://doi.org/10.1182/blood-2016-12-758250
2. Bystrykh L V, Belderbos ME (2016) Clonal Analysis of Cells with Cellular Barcoding: When Numbers and Sizes Matter. In: Turksen K (ed) Stem Cell Heterogeneity: Methods and Protocols. Springer New York, New York, NY, pp 57–89
3. Verovskaya E, Broekhuis MJC, Zwart E, et al (2013) Heterogeneity of young and aged murine hematopoietic stem cells revealed by quantitative clonal analysis using cellular barcoding. Blood 122:523 LP – 532. https://doi.org/10.1182/blood-2013-01-481135
4. R Core Team (2019) R: A Language and Environment for Statistical Computing
5. Larsson J (2019) eulerr: Area-Proportional Euler and Venn Diagrams with Ellipses
6. Wickham H (2016) ggplot2: Elegant Graphics for Data Analysis. Springer-Verlag New York
